# Structural Investigation of the Synthesized Few-Layer Graphene from Coal under Microwave

**DOI:** 10.3390/nano12010057

**Published:** 2021-12-26

**Authors:** Faridul Islam, Arash Tahmasebi, Behdad Moghtaderi, Jianglong Yu

**Affiliations:** 1 Department of Chemical Engineering, School of Engineering, The University of Newcastle, Callaghan, NSW 2308, Australia; faridul.islam@uon.edu.au (F.I.); arash.tahmasebi@newcastle.edu.au (A.T.); behdad.moghtaderi@newcastle.edu.au (B.M.); 2 Monash Research Institute of Science and Technology (Suzhou Industrial Park), Southeast University—Monash University Joint Graduate School, Suzhou 215000, China

**Keywords:** few-layer graphene, coal, Raman spectroscopy, catalyst, microwave

## Abstract

This study focused on the structural investigation of few-layer graphene (FLG) synthesis from bituminous coal through a catalytic process under microwave heat treatment (MW). The produced FLG has been examined by Raman spectroscopy, XRD, TEM, and AFM. Coal was activated using the potassium hydroxide activation process. The FLG synthesis processing duration was much faster requiring only 20 min under the microwave radiation. To analyse few-layer graphene samples, we considered the three bands, i.e., D, G, and 2D, of Raman spectra. At 1300 °C, the P10% Fe sample resulted in fewer defects than the other catalyst percentages sample. The catalyst percentages affected the structural change of the FLG composite materials. In addition, the Raman mapping showed that the catalyst loaded sample was homogeneously distributed and indicated a few-layer graphene sheet. In addition, the AFM technique measured the FLG thickness around 4.5 nm. Furthermore, the HRTEM images of the P10% Fe sample contained a unique morphology with 2–7 graphitic layers of graphene thin sheets. This research reported the structural revolution with latent feasibility of FLG synthesis from bituminous coal in a wide range.

## 1. Introduction

In recent years, the focus on graphene in the research and commercial sectors has increased remarkably worldwide due to its novel properties such as thermodynamically stability, transparency, and higher mechanical strength and for its potential applications in several fields, such as in sensors [1], batteries [2], ultrafast photodetectors [3], transparent electrodes [4], and advanced nanocomposites [5]. Graphene is a sp^2^-bonded monolayer carbon atom arranged in a 2D honeycomb frame. It displays unique electronic properties with high mobility and transportation [6,7]. SLG has a zero bandgap structure, which affects its potential electronics applications and optical performance [8]. In contrast, FLG has attracted more commercial applications due to the potential to control electronic states using interlayer connections [9]. Currently, the synthesis of SLG and FLG can be achieved using several methods such as the liquid exfoliation of graphite [10], epitaxy growth in an ultrahigh vacuum [11], mechanical cleavage [12], chemical vapour deposition (CVD) [13], chemical reduction [14], etc. Each of the techniques has some drawbacks that are not feasible for the mass production of graphene [15]. However, these techniques are expensive, and the production processes also contain toxic chemicals and long processing times. Thus, there is a need to establish a cost-effective process that is a fast and simple technique and has scalable production [16]. Among the various methods, the CVD procedure is the most promising technique to make a high-quality mono- or few-layer graphene with large coverage areas using catalytic substrates and hydrocarbon gases [17,18].

In addition, coal is an inexpensive and plentiful material worldwide, leading to alternative applications [19]. Coals and food waste have also been used as solid carbon sources instead of gaseous hydrocarbons to produce graphene [20]. Moreover, the graphite materials are used as a precursor to synthesize the graphene oxide, a time-consuming modified Hummers reduction process [21]. These days, MW heating plays a significant role in processing many materials. It has an interaction capacity with materials at a molecular level, and its polarization plays an important role in heating materials [22]. Moreover, the heating equality depends on the microwave extrinsic properties (MW frequency, cavity, and power amplitude) and sample intrinsic properties (size and shape). The sample shape also plays a crucial role in MW heating consistency and heating rate [23,24]. The MW-assisted graphene synthesis provides extra benefits compared to the conventional synthesis methods. It has also been used to synthesise many graphene-derivative products, such as reduced graphene oxide (rGO) [25], graphene nanoribbons (GNRs) [26], and graphene oxide (GO) [27], which are frequently used in supercapacitors and battery applications for energy storage [28,29].

Carbon and its derivatives such as graphite, carbon nanotubes (CNTs), and graphene are also synthesized using MW radiation. Carbon materials have an excellent MW absorption capacity due to the delocalisation of pi (π) electrons from the sp^2^ hybridized carbon atom [30]. However, the CVD method synthesises the multilayer graphene, which has a multiplicative effect with a better diffusion barrier than SLG [31]. The properties of FLGs, such as their electronic band structure, depend on the samples due to numerous crystallographic stacking orders. Therefore, the FLG is used widely in various sectors due to its potential capacity to control the electronic states through an interlayer interaction [32]. Raman spectroscopy has been extensively used to analyse graphite materials at the electronic band arrangements and vibrational ranges using the double-resonance Raman scattering mechanism [33]. The principal features of the Raman spectra such as the D, G, and 2D bands alter the location, which is related to the structural and electronic properties of composite materials [34].

With the increasing benefits of graphene-based nanotechnologies, there is a high demand to develop the production techniques for high-quality FLG. However, the industrial sector also requires cost- and time-efficient graphene production methods with a way to control layer thickness. The present work synthesized FLG from bituminous coal using a catalytic graphitization process (iron (III) nitrate nonahydrate) with MW heating for 20 min at 1300 °C. Raman spectra and mapping were applied to determine numerous aspects of the graphene produced. The fabricated FLG samples were analysed using XRD, Raman spectroscopy, TEM, and AFM.

## 2. Materials and Methods

### 2.1. Materials and Sample Preparation

The Australian bituminous coal was activated using a potassium hydroxide (KOH) activation process to increase the porosity of FLG composite materials. The raw coal was heated at a fixed rate of N_2_ flow (200 mL/min) for 30 min by the electric furnace (Carbolite-VST 12/300, UK) at 600 °C (10 °C/min) to remove the volatile matter and other lightweight compounds. The sample was then ground and sieved to <63 μm and was consequently activated by the potassium hydroxide (KOH) at a ratio of 1:4 (coal:KOH) followed by carbonization at a temperature of 900 °C [35]. According to sample weight percentage, the Fe(NO_3_)_3_·9H_2_O (≥99.95%) (Merck, Germany) catalyst was mixed overnight with a sample at 2%, 5%, 10%, and 20%, and then 0.1 M ammonia solution (25%) was used individually and stirred for 10 min. Finally, the slurry was washed with distilled water, filtered, and dried at 110 °C. The KOH-modified, iron-loaded porous samples were denoted as P2% Fe, P5% Fe, P10% Fe, and P20% Fe.

### 2.2. Synthesis of the FLG Materials

A custom modified quartz reactor and B-type (up to 1600 °C) thermocouple was used inside a MW oven (Tangshan microwave thermal instrument CO. Ltd.; Beijing, China) to synthesize and measure the sample temperatures, briefly explained in our previous study [36]. In addition, a 3 g sample for each experiment was treated continuously with N_2_ gas flow (200 mL/min) through the quartz reactor for 30 min prior to the start of the experiment. The catalyst-loaded samples with different catalyst percentages, from 2% to 20%, were graphitized at a temperature of 1300 °C for 20 min with the same N_2_ flow rate.

### 2.3. Structural Analysis of the FLG Materials

The crystallinity of FLG composite materials was investigated with a Horiba XploRA PLUS Raman microscope (HORIBA France) with a 532 nm wavelength, which indicated the three different bands such as D ≈ 1350 cm^−1^, G ≈ 1580 cm^−1^, and 2D ≈ 2700 cm^−1^, which are used to quantify the graphitization degree of samples. The thicknesses and cross-sectional images of the FLG samples were measured using an Asylum Research Cypher AFM (Oxford Instruments, CA). In addition, the texture of the FLG samples was scrutinized by the transmission electron microscope (JEOL TEM 2100) at 200 kV. BET was used to calculate the surface area of the materials. The X-ray diffraction (XRD) (Bruker, Berlin, Germany) was used for crystallinity in a wide range of 10° to 80°. Furthermore, the sample crystal sizes (La) and heights (Lc) with an interlayer spacing (d_002_) were measured by the Scherrer and Bragg equation. In addition, their g factor values were determined by the Marie and Meiring rules [37]. The Raman mappings were detected by an electron multiplication CCD camera (EMCCD). The confocal imaging was 0.5 μm. The fine powder samples were used to focus the laser on. In addition, three samples were used for each material. The repeat scans and acquisition times were dependent on the signal-to-noise ratios of the samples. The Raman spectra were focussed through a 100× objective microscope and were optimized at the highest spectral counts.

## 3. Results and Discussion

### 3.1. Morphological Analysis of the FLG Materials

**Figure 1:**
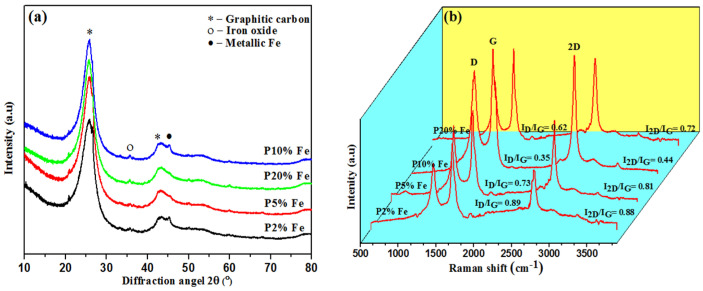
(**a**) XRD and (**b**) Raman spectra of the Fe-loaded samples.

XRD diffraction profiles of the Fe catalyst-loaded samples at a temperature of 1300 °C are shown in Figure 1a. The two distinctive peak positions were at around 26° and 42.5°, respectively, which can be assigned to 002 and 100 reflections of graphite, suggesting a typical graphitic structure [38]. The highest intensity peak was obtained for the P10% Fe sample. The P20% Fe sample’s intensity was 26°, which was less than the P10% Fe sample, due to having a more amorphous carbon structure. The metallic Fe present at around 45.2° was formed by the degradation of the iron oxide during the graphitization period. It is also represented as a nucleus to form metallic iron’s graphitic layers [39]. Table 1 shows the structural parameters measured with the XRD using the Scherrer and Bragg and Marie and Meiring equations, such as the particle size (La), thickness (Lc), interlayer distance (d_002_), and g factor [37]. The results showed that the crystal sizes for the P2% Fe of 1.42 nm, P5% Fe of 1.50 nm, P10% Fe of 1.98 nm, and P20% Fe of 1.79 nm, as well as the thicknesses for the P2% Fe of 3.07 nm, P5% Fe of 3.24 nm, P10% Fe of 4.27 nm, and P20% Fe of 3.85 nm. The P10% Fe catalyst obtained the largest sizes and thicknesses. The results for the P20% Fe sample were reduced due to the catalytic agglomeration effect and having a more disordered structure. The values for the degree of graphitization (g factor) were 85.9%, 90.7%, 96.8%, and 92.2%, respectively (see Table 1). The highest graphitization was achieved by the P10% Fe sample, with 96.8%. Furthermore, it was found that the MW catalytic graphitization also assisted in reducing the interlayer spacing distances of the samples [40]. The interlayer distance (d_002_) values were very close for all samples, showing that the crystal structures changed from disordered to ordered structures. The interlayer distance (d_002_) values for the P2% to P20% Fe samples were in the range of 0.3357–0.3366 nm, which suggested a turbostratic structure due to larger values than those of graphite (0.335 nm) [41]. At a heating temperature of 1300 °C, the results showed that the P10% Fe samples had d_002_ values of 0.3357, which was close to the structure of graphitic carbon. Moreover, the highest graphitization value (g factor) found was for the P10% Fe sample (96.8%), which corresponded with the degradation of numerous aliphatic chains and functional groups [42].

The adsorption–desorption isotherm measured the textural properties of composite samples. The P10% Fe sample-specific surface area was 315.45 m^2^g^−1^, which was higher than the other percentages (see Table 1). It has been found that KOH-activated samples have higher surface areas than steam-activated single and dual catalyst loading samples (109.3 m^2^g^−1^ and 175.61m^2^g^−1^) [36,43].

Raman spectroscopy has broadly been applied to scrutinize carbon nanostructures because it is a nondestructive tool and is sensitive enough to determine molecular bonding and geometric structures [44]. The Raman spectra for the graphene and graphite were screened to 1000–3000 cm^−1^ area and showed three prominent peaks such as D, G, and 2D. At 1350 cm^−1^, the D peak was attributed to defects present in the samples, and its intensity also indicated the amount of disordered structure. The G peak (1580 cm^−1^) corresponded to the sp^2^-hybridization, and the peak position and intensity are influential in determining the f graphene layer numbers [45]. The 2D peak (2700 cm^−1^) arose from the two double resonance phenomena with equal but opposite wave vector phonons and is the second most prominent graphite band. Ferrari and his co-workers investigated that the multilayer graphene electronic band structure has changed for the 2D band position, intensity, and shape [46].

**Figure 2:**
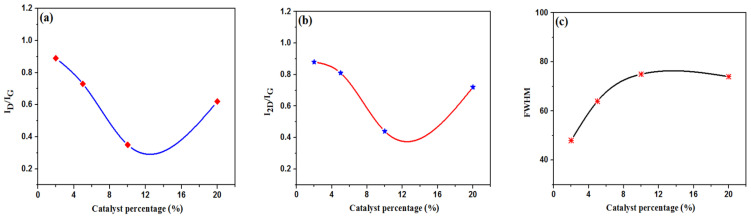
Results for: (**a**) I_D_/I_G_; (**b**) I_2D_/I_G_; and (**c**) FWHM of the 2D band function for the different percentages of the Fe catalyst.

The Raman spectra of P2% to P20% are recorded in Figure 1b. The disordered and ordered structures are represented by the D and G bands in the Raman spectra, and the I_D_/I_G_ ratio showed the graphitization degree. In addition, the I_D_/I_G_ results showed P2% Fe was 0.89, P5% Fe was 0.73, P10% Fe was 0.35, and P20% Fe was 0.862, with the P10% Fe sample containing the lowest value (0.35) (Figure 2a). It showed that the homogenous and continuous graphene carbon nanostructures were formed in the P10% Fe sample [47]. However, the degree of graphitization value for the P20% was higher than the P10% sample due to the catalytic aggregation, and a high amount of disordered structures were present in the sample [48].

Another prominent 2D band was detected at 2700 cm^−1^, which is frequently used to confirm the thickness of the graphene. In addition, the 2D peak is highly influential because the duel- or triple-resonance produces a photoexcited electron–hole pair with a different energy. The layer numbers changed with the nature of the 2D peak, which indicated that the electronic band structure changed. Moreover, the 2D band positions lifted to a higher number as percentages of the catalyst were increased, which corresponded to the increase in the number of graphene layers [49] (see Table 2). Furthermore, the I_2D_/I_G_ ratio determined the number of graphene layers. The lowest ratio for the I_2D_/I_G_ was 0.44, which was observed for the P10% Fe sample, and it corresponded to the FLG structure (Figure 2b) [47]. Moreover, the I_2D_/I_D_ ratio signified the overall crystalline structures of all samples analysed, shown in Table 2. The P10% Fe sample obtained the highest I_2D_/I_D_ value of 1.24, which indicated a longer graphitic structure [50]. The full width half maximum (FWHM) of the 2D band measured the number of graphene layers, which is shown in Figure 2c [51]. The FWHM values for the different percentages of catalyst were 48 ± 0.15 cm^−1^ for P2% Fe, 64 ± 0.17 cm^−1^ for P5% Fe, 75 ± 0.26 cm^−1^ for P10% Fe, and 74 ± 0.24 cm^−1^ for P20% Fe, which suggest the formation of FLG, which is supported by the findings in other studies [47,52] (Table 2).

**Figure 3:**
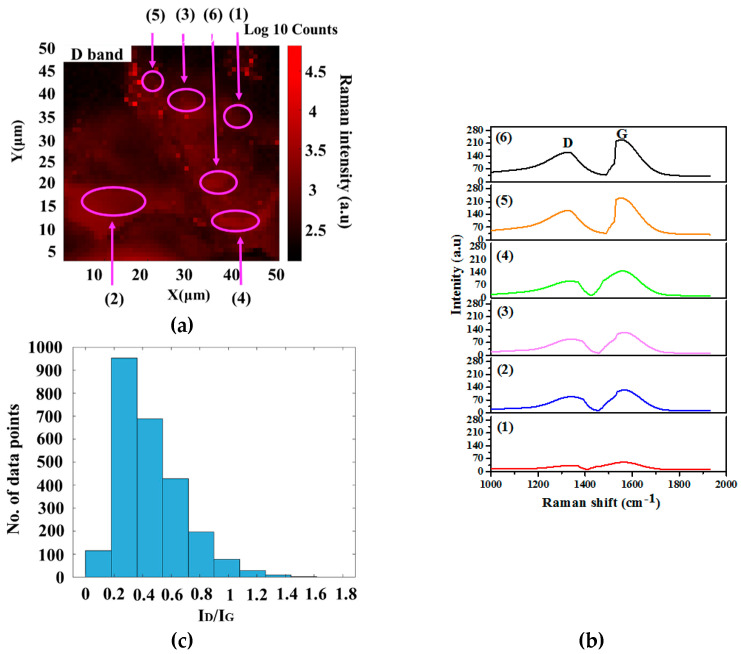
Raman mapping for the D band and a histogram of the I_D_/I_G_ ratio of the P10% sample.

**Figure 4:**
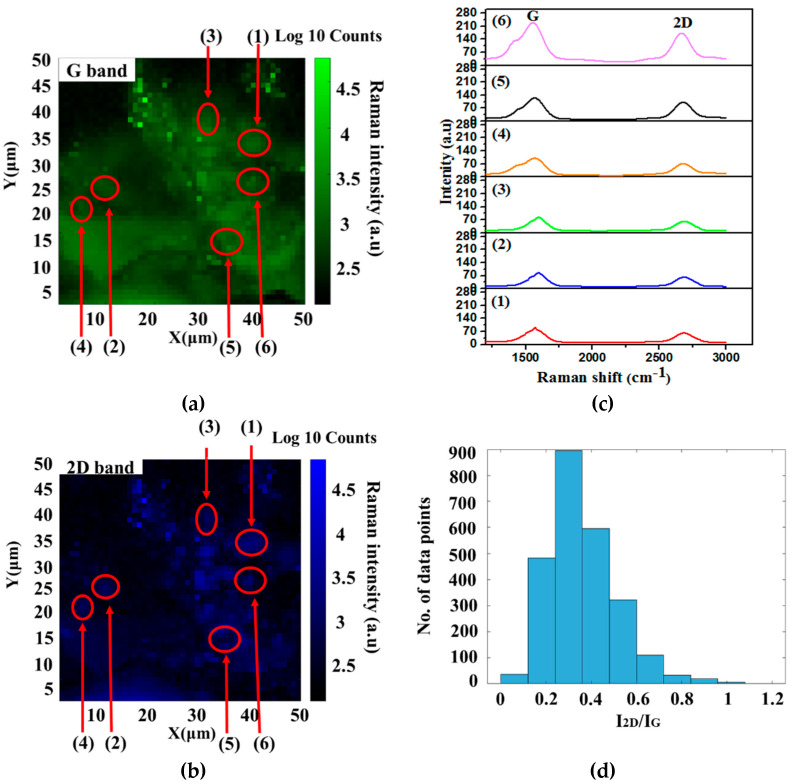
G and 2D bands Raman mapping with a histogram of the I_2D_/I_G_ ratios of P10% sample.

The Raman mapping was applied to determine the thicknesses of graphene [53]. The results of the Raman intensity mapping for the P10% Fe sample on a quartz substrate are represented in Figure 3 and Figure 4. The peak area locations for the three (3) bands were 1350 cm^−1^, 1580 cm^−1^, and 2700 cm^−1^, which are displayed in red, green, and blue, respectively. The mapping indicated that the sheets were homogeneously spread on the quartz substrate (Figure 3 and Figure 4). The brighter zones of the D band showed the sample’s high intensity (Figure 3). In addition, the defects were larger in the perkier region, ascribed to edge defects. The degree of graphitization ratios (I_D_/I_G_) for the points (1), (2), (3), (4), (5), and (6) were 0.15, 0.19, 0.28, 0.32, 0.34, and 0.37 respectively. The defect ratios for points (1)–(3) were 0.15–0.28. However, points (4)–(6) were 0.32–0.37, indicating that the lower height of the D band was correlated with the high quality of the FLG, and the other findings are supported. Furthermore, the D band arises from the red boundary. The histogram of I_D_/I_G_ ratio of 600 data points was obtained using the Raman mapping. It is clear from the histogram graph that the I_D_/I_G_ ratio of all samples remained the same value, and the low intensity of the D band represents the FLG obtained.

The Raman intensity mapping at various points for the G and 2D are presented in Figure 4. The G and second order 2D bands rise from the green and navy-blue boundaries. Both bands have brighter areas and defect zones. The I_2D_/I_G_ intensity ratio indicated the layer numbers of the graphene sheets, which are obtained from the Raman mapping. The I_2D_/I_G_ intensity ratios were around 78% for points (1)–(4), 0.45 to 0.51. However, for points (5) and (6), the I_2D_/I_G_ values were 0.68 and 0.77, respectively, which was remarkably high and indicated that the FLG formed. The histogram results also correlated with the I_2D_/I_G_ ratios exposed in Figure 4 [54].

### 3.2. AFM and TEM Examine the FLG Materials

**Figure 5:**
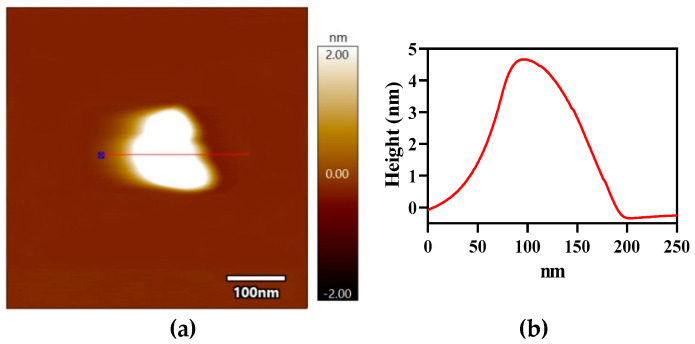
AFM image of the P10% Fe sample with corresponding line profile.

Morphological changes of the P10% Fe sample were examined using AFM, and the results are displayed in Figure 5. The thickness of the graphene was measured with the AFM technique by studying the upper view image and the cross-sectional of the composite materials [55]. Figure 5 shows that the graphene sheets were sound-exfoliated and that the average sheet thickness was around 4.5 nm, which indicated the existence of FLG sheets [56].

**Figure 6:**
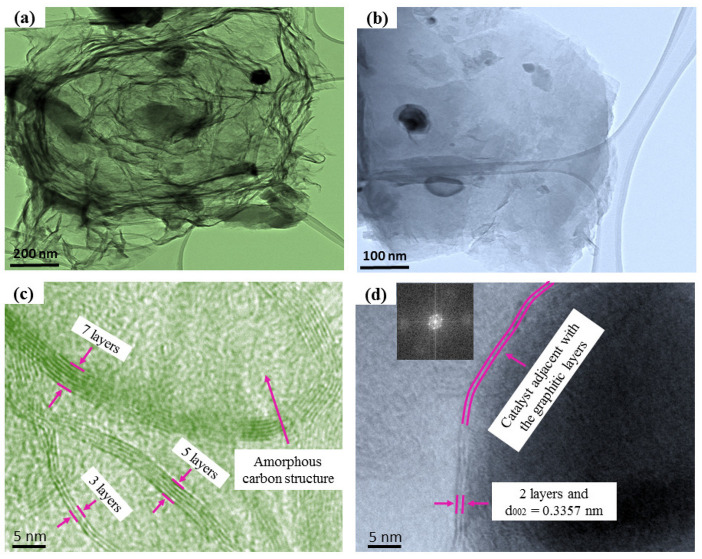
(**a**) and (**b**): TEM of the P10% sample with different magnifications. (**c**,**d**): the HRTEM showing the FLG sheets (and the FFT image in the inset of (**d**)).

The structure and morphology were further investigated by employing TEM to confirm the FLG materials. The TEM micrograph for the P10% Fe sample showed FLG-like nanosheets at numerous magnifications on a lacy carbon grid (Figure 6a,b). The graphene plate was thin, and the plane with the wrinkles formed the back foldaway and touched the edge because of the transfer method. A transition metal such as Fe could reduce the melting temperature of the Fe and carbon due to their d-electron configuration and ionization abilities. The amorphous carbon melted over the catalyst at the supersaturation point of Fe–C. As a result, the graphitic layer was formed due to the dissolution and precipitation mechanism, whereas the catalysts worked as nuclei [57,58]. In addition, the Fe–C eutectic point was 1148 °C, which was recorded from the Fe–C phase diagram [59]. The high-resolution HRTEM images of the P10% Fe sample in the selected regions highlighted the distorted nanosheets containing around 2–7 layers (Figure 6c,d). The KOH-activated sample achieved a fewer number of layers of graphene compared to the steam-activated sample [43].

The HRTEM images also identified that the interlayer distance was around 0.34 nm (Figure 6d), which resembled the plane structure of FLG (002). It also agreed with the XRD data, as mentioned above. Furthermore, the fast Fourier transform (FFT) image (inset of Figure 6d) showed the hexagonal spot configuration. It confirmed the six-fold symmetry graphene features with the crystalline nature of the materials [60].

## 4. Conclusions

The coal-based synthesis of FLG was fabricated through potassium hydroxide modification with an MW graphitization technique. It was found that the catalyst loading, microwave temperature, and potassium hydroxide activation played significant roles in manufacturing the FLG composite materials. The synthesis of the P10% Fe-loaded sample at a heating temperature of 1300 °C created a unique morphology with 2–7 graphitic layers and lower defect levels, demonstrating that more regular and continuous thin sheets of graphene were formed. The catalyst loading percentages determined the structural change. The results of the TEM analyses revealed the sheets of the synthesized graphene (P10% Fe). The Raman mapping measurements showed that at 1300 °C, the P10% Fe-loaded sample was homogeneously distributed. The average detected I_D_/I_G_ ratio was around 0.35, and the highest I_2D_/I_G_ values were 0.68–0.77, indicating a sheet of FLG. Moreover, the AFM technique measured that the thickness of the FLG was around 4.5 nm. The few-layer graphene has several potential applications in many fields such as energy storage (lithium-ion battery and supercapacitor), biomedical applications to targeted drug delivery, sensors, membranes, and the electronics arena.

## Figures and Tables

**Table 1:** Structural properties at the different percentages of Fe catalyst.

Catalyst Loading (% of Fe)/Parameters	P2	P5	P10	P20
Interlayer spacing (d_002_) (nm) ^1^	0.3366	0.3362	0.3357	0.3361
Crystal size (Lc) (nm) ^1^	1.42	1.50	1.98	1.79
In-plane crystal thickness (La) (nm)	3.07	3.24	4.27	3.85
I_D_/I_G_	0.89	0.73	0.35	0.62
Surface area (m^2^g^−1^)	221.08	275.74	315.45	282.36
“g” factor (%) ^2^	85.9	90.7	96.8	92.1

^1^ Matches to the (002) graphitic plane, which is calculated from XRD data. ^2^ The degree of graphitization value measured from g = (0.344–d_002_)/(0.344–0.3354).

**Table 2:** Raman spectroscopy results of P2% to P20% Fe-loaded sample.

Fe (%) Loading/Parameters	P2	P5	P10	P20
Position of 2D (cm^−1^)	2698 ± 0.19	2699 ± 0.21	2702 ± 0.24	2703 ± 0.22
I_2D_/I_G_	0.88	0.81	0.44	0.72
I_2D_/I_D_	0.98	1.11	1.24	1.17
FWHM (2D)	48 ± 0.15	64 ± 0.17	75 ± 0.26	74 ± 0.24

## Data Availability

Data contained within this article.

## References

[1] Díaz P., González Z., Santamaría R., Granda M., Menéndez R., Blanco C. (2016). Enhancing energy density of carbon-based supercapacitors using Prussian Blue modified positive electrodes. Electrochim. Acta.

[2] Dobrota A.S., Pašti I.A., Mentus S.V., Johansson B., Skorodumova N.V. (2017). Functionalized graphene for sodium battery applications: The DFT insights. Electrochim. Acta.

[3] Xia F., Mueller T., Lin Y.-M., Valdes-Garcia A., Avouris P. (2009). Ultrafast graphene photodetector. Nat. Nanotechnol..

[4] Kim K.S., Zhao Y., Jang H., Lee S.Y., Kim J.M., Kim K.S., Ahn J.-H., Kim P., Choi J.-Y., Hong B.H. (2009). Large-scale pattern growth of graphene films for stretchable transparent electrodes. Nature.

[5] Sierra U., Mercado A., Cuara E., Barriga-Castro E.D., Cortés A., Gallardo-Vega C., Fernández S. (2020). Coke-derived few layer graphene-like materials by mild planetary milling exfoliation. Fuel.

[6] Geim A.K., Novoselov K.S. (2010). The rise of graphene. Nanoscience and Technology: A Collection of Reviews from Nature Journals.

[7] Tse W.-K., Hwang E., Das Sarma S. (2008). Ballistic hot electron transport in graphene. Appl. Phys. Lett..

[8] Neto A.C., Guinea F., Peres N.M., Novoselov K.S., Geim A.K. (2009). The electronic properties of graphene. Rev. Mod. Phys..

[9] Castro E.V., Novoselov K., Morozov S., Peres N., Dos Santos J.L., Nilsson J., Guinea F., Geim A., Neto A.C. (2007). Biased bilayer graphene: Semiconductor with a gap tunable by the electric field effect. Phys. Rev. Lett..

[10] Hernandez Y., Nicolosi V., Lotya M., Blighe F.M., Sun Z., De S., McGovern I., Holland B., Byrne M., Gun’Ko Y.K. (2008). High-yield production of graphene by liquid-phase exfoliation of graphite. Nat. Nanotechnol..

[11] Berger C., Song Z., Li T., Li X., Ogbazghi A.Y., Feng R., Dai Z., Marchenkov A.N., Conrad E.H., First P.N. (2004). Ultrathin epitaxial graphite: 2D electron gas properties and a route toward graphene-based nanoelectronics. J. Phys. Chem. B.

[12] Novoselov K.S., Geim A.K., Morozov S.V., Jiang D., Zhang Y., Dubonos S.V., Grigorieva I.V., Firsov A.A. (2004). Electric field effect in atomically thin carbon films. Science.

[13] Muñoz R., Gómez-Aleixandre C. (2013). Review of CVD synthesis of graphene. Chem. Vap. Depos..

[14] Abdolhosseinzadeh S., Asgharzadeh H., Kim H.S. (2015). Fast and fully-scalable synthesis of reduced graphene oxide. Sci. Rep..

[15] Galindo B., Alcolea S.G., Gómez J., Navas A., Murguialday A.O., Fernandez M.P., Puelles R. (2014). Effect of the number of layers of graphene on the electrical properties of TPU polymers. IOP Conf. Ser. Mater. Sci. Eng..

[16] Krane N. Preparation of Graphene. Selected Topics in Physics: Physics of Nanoscale Carbon 2011. https://www.physik.fu-berlin.de/einrichtungen/ag/ag-reich/lehre/Archiv/ss2011/docs/Nils_Krane-Handout.pdf.

[17] Li X., Cai W., An J., Kim S., Nah J., Yang D., Piner R., Velamakanni A., Jung I., Tutuc E. (2009). Large-area synthesis of high-quality and uniform graphene films on copper foils. Science.

[18] Reina A., Jia X., Ho J., Nezich D., Son H., Bulovic V., Dresselhaus M.S., Kong J. (2009). Large area, few-layer graphene films on arbitrary substrates by chemical vapor deposition. Nano Lett..

[19] Wang G., Zhang L., Zhang J. (2012). A review of electrode materials for electrochemical supercapacitors. Chem. Soc. Rev..

[20] Vijapur S.H., Wang D., Botte G.G. (2013). Raw coal derived large area and transparent graphene films. ECS Solid State Lett..

[21] Bi H., Wan S., Cao X., Wu X., Zhou Y., Yin K., Su S., Ma Q., Sindoro M., Zhu J. (2019). A general and facile method for preparation of large-scale reduced graphene oxide films with controlled structures. Carbon.

[22] Menéndez J., Arenillas A., Fidalgo B., Fernández Y., Zubizarreta L., Calvo E.G., Bermúdez J.M. (2010). Microwave heating processes involving carbon materials. Fuel Process. Technol..

[23] Fung D.Y., Cunningham F. (1980). Effect of microwaves on microorganisms in foods. J. Food Prot..

[24] Soto-Reyes N., Temis-Pérez A.L., López-Malo A., Rojas-Laguna R., Sosa-Morales M.E. (2015). Effects of shape and size of agar gels on heating uniformity during pulsed microwave treatment. J. Food Sci..

[25] Jiang F., Yu Y., Wang Y., Feng A., Song L. (2017). A novel synthesis route of graphene via microwave assisted intercalation-exfoliation of graphite. Mater. Lett..

[26] Amiri A., Sadri R., Shanbedi M., Ahmadi G., Kazi S., Chew B., Zubir M.N.M. (2015). Synthesis of ethylene glycol-treated graphene nanoplatelets with one-pot, microwave-assisted functionalization for use as a high performance engine coolant. Energy Convers. Manag..

[27] Zhu Y., Murali S., Stoller M.D., Velamakanni A., Piner R.D., Ruoff R.S. (2010). Microwave assisted exfoliation and reduction of graphite oxide for ultracapacitors. Carbon.

[28] Liu T., Chai H., Jia D., Su Y., Wang T., Zhou W. (2015). Rapid microwave-assisted synthesis of mesoporous nimoo4 nanorod/reduced graphene oxide composites for high-performance supercapacitors. Electrochim. Acta.

[29] Zhou X., Shi J., Liu Y., Su Q., Zhang J., Du G. (2014). Microwave-assisted synthesis of hollow cuo–Cu2O nanosphere/graphene composite as anode for lithium-ion battery. J. Alloys Compd..

[30] Kim T., Lee J., Lee K.-H. (2014). Microwave heating of carbon-based solid materials. Carbon Lett..

[31] Prasai D., Tuberquia J.C., Harl R.R., Jennings G.K., Bolotin K.I. (2012). Graphene: Corrosion-inhibiting coating. ACS Nano.

[32] Yao W., Xiao D., Niu Q. (2008). Valley-dependent optoelectronics from inversion symmetry breaking. Phys. Rev. B.

[33] Thomsen C., Reich S. (2000). Double resonant Raman scattering in graphite. Phys. Rev. Lett..

[34] Malard L., Pimenta M.A., Dresselhaus G., Dresselhaus M. (2009). Raman spectroscopy in graphene. Phys. Rep..

[35] Govind Raj K., Joy P.A. (2017). Role of localized graphitization on the electrical and magnetic properties of activated carbon. J. Am. Ceram. Soc..

[36] Islam F., Tahmasebi A., Wang R., Yu J. (2021). Structure of Coal-Derived Metal-Supported Few-Layer Graphene Composite Materials Synthesized Using a Microwave-Assisted Catalytic Graphitization Process. Nanomaterials.

[37] Yeh T.-S., Wu Y.-S., Lee Y.-H. (2011). Graphitization of unburned carbon from oil-fired fly ash applied for anode materials of high power lithium ion batteries. Mater. Chem. Phys..

[38] Wang R., Lu G., Qiao W., Yu J. (2016). Catalytic graphitization of coal-based carbon materials with light rare earth elements. Langmuir.

[39] Xiong W., Zhou Y.S., Hou W.J., Guillemet T., Silvain J.-F., Gao Y., Lahaye M., Lebraud E., Xu S., Wang X. (2015). Solid-state graphene formation via a nickel carbide intermediate phase. RSC Adv..

[40] Kim T., Lee J., Lee K.-H. (2016). Full graphitization of amorphous carbon by microwave heating. RSC Adv..

[41] Inagaki M. (2000). New Carbons-Control of Structure and Functions.

[42] Kim Y.-J., Yang H., Yoon S.-H., Korai Y., Mochida I., Ku C.-H. (2003). Anthracite as a candidate for lithium ion battery anode. J. Power Sources.

[43] Islam F., Wang J., Tahmasebi A., Wang R., Moghtaderi B., Yu J. (2021). Microwave-Assisted Coal-Derived Few-Layer Graphene as an Anode Material for Lithium-Ion Batteries. Materials.

[44] Ferrari A.C., Basko D.M. (2013). Raman spectroscopy as a versatile tool for studying the properties of graphene. Nat. Nanotechnol..

[45] Khan M.F., Iqbal M.Z., Iqbal M.W., Eom J. (2014). Improving the electrical properties of graphene layers by chemical doping. Sci. Technol. Adv. Mater..

[46] Ferrari A.C., Meyer J.C., Scardaci V., Casiraghi C., Lazzeri M., Mauri F., Piscanec S., Jiang D., Novoselov K.S., Roth S. (2006). Raman spectrum of graphene and graphene layers. Phys. Rev. Lett..

[47] Prekodravac J., Marković Z., Jovanović S., Holclajtner-Antunović I., Pavlović V., Todorović-Marković B. (2016). Raman spectroscopy study of graphene thin films synthesized from solid precursor. Opt. Quantum Electron..

[48] Bîru E.I., Iovu H. (2018). Graphene nanocomposites studied by Raman spectroscopy. Raman Spectrosc..

[49] Ni Z., Wang Y., Yu T., Shen Z. (2008). Raman spectroscopy and imaging of graphene. Nano Res..

[50] Sharma B., Schumann T., de Oliveira M.H., Lopes J.M.J. (2017). Controlled synthesis and characterization of multilayer graphene films on the C-face of silicon carbide. Phys. Status Solidi.

[51] Kim K., Coh S., Tan L.Z., Regan W., Yuk J.M., Chatterjee E., Crommie M., Cohen M.L., Louie S.G., Zettl A. (2012). Raman spectroscopy study of rotated double-layer graphene: Misorientation-angle dependence of electronic structure. Phys. Rev. Lett..

[52] Ferrari A.C. (2007). Raman spectroscopy of graphene and graphite: Disorder, electron–phonon coupling, doping and nonadiabatic effects. Solid State Commun..

[53] Graf D., Molitor F., Ensslin K., Stampfer C., Jungen A., Hierold C., Wirtz L. (2007). Raman mapping of a single-layer to double-layer graphene transition. Eur. Phys. J. Spec. Top..

[54] Gayathri S., Jayabal P., Kottaisamy M., Ramakrishnan V. (2014). Synthesis of few layer graphene by direct exfoliation of graphite and a Raman spectroscopic study. Aip Adv..

[55] Ramalingam P., Pusuluri S.T., Periasamy S., Veerabahu R., Kulandaivel J. (2013). Role of deoxy group on the high concentration of graphene in surfactant/water media. RSC Adv..

[56] Peng K.-J., Lin Y.-H., Wu C.-L., Lin S.-F., Yang C.-Y., Lin S.-M., Tsai D.-P., Lin G.-R. (2015). Dissolution-and-reduction CVD synthesis of few-layer graphene on ultra-thin nickel film lifted off for mode-locking fiber lasers. Sci. Rep..

[57] Ōya A., Ōtani S. (1979). Catalytic graphitization of carbons by various metals. Carbon.

[58] Derbyshire F., Presland A., Trimm D. (1975). Graphite formation by the dissolution—precipitation of carbon in cobalt, nickel and iron. Carbon.

[59] Bystrzejewski M. (2011). Synthesis of carbon-encapsulated iron nanoparticles via solid state reduction of iron oxide nanoparticles. J. Solid State Chem..

[60] Zan R. (2013). Microscopy and Spectroscopy of Graphene: Atomic Scale Structure and Interaction with Foreign Atom Species.

